# The performance of BD FACSPresto™ for CD4 T-cell count, CD4% and hemoglobin concentration test in Ethiopia

**DOI:** 10.1371/journal.pone.0176323

**Published:** 2017-04-27

**Authors:** Gebremedhin Gebremicael, Yohanes Belay, Fitsum Girma, Yemane Abreha, Atsbeha Gebreegziabxier, Simret Tesfaye, Zelalem Messele, Yibeltal Assefa, Bahrie Bellete, Desta Kassa, Lara Vojnov

**Affiliations:** 1 HIV and TB diseases research directorate, Ethiopian Public Health Institute (EPHI), Addis Ababa, Ethiopia; 2 Clinton Health Access Initiative, Inc. (CHAI), Addis Ababa, Ethiopia; 3 Clinton Health Access Initiative, Boston, Massachusetts, United States of America; National Health Laboratory Service, SOUTH AFRICA

## Abstract

**Introduction:**

In Ethiopia, CD4+ T-cell counting is still required for all patients at baseline before antiretroviral therapy (ART) and to determine eligibility and follow-up of opportunistic infection prophylaxis. However, access to CD4+ T cell count in rural health facilities remains a major challenge in Ethiopia like other resource-limited settings.

**Methodology:**

Both capillary and venous blood was drawn from each of 325 study participant recruited in Addis Ababa and surroundings. The CD4+ T-cell count, CD4%, and hemoglobin (Hgb) were tested at one of the four study health facilities using capillary blood and BD FACSPresto™ device. These tests were also done at the national HIV reference laboratory, using venous blood with BD FACSCalibur™, Sysmex XT-1800i™, and BD FACSPresto™.

**Results:**

BD FACSPresto™ had an absolute mean bias of -13.3 cells/ul (-2.99%) and 28.3 cells/μl (6.4%) using venous and capillary blood, respectively, compared with BD FACSCalibur™. The absolute CD4 assay on the BD FACSPresto™ had a regression coefficient (R^2^) of 0.87 and 0.92 using capillary blood and venous blood samples, respectively, compared with BD FACSCalibur™. The percentage similarity of the BD FACSPresto™ using capillary and venous blood was 105.2% and 99.3%, respectively. The sensitivity of the FACSPresto™ using threshold of 500 cells/μl for ART eligibility using capillary and venous blood was 87.9 and 94.3%, while the specificity was 91.4 and 83.8%, respectively. Furthermore, the BD FACSPresto™ had an absolute mean bias of -0.2 dl/μl (0.0%) (95% LOA: -1.7, 1.3) and -0.59 dl/μl (0.1%) (95% LOA: -1.49, 0.31) for Hgb using capillary and venous blood compared with the Sysmex XT-1800i™, respectively.

**Conclusion:**

Our results showed acceptable agreement between the BD FACSPresto™ and BD FACSCalibur™ for CD4+ T-cell counting and CD4%; and between the BD FACSPresto™ and Sysmex XT-1800i™for measuring Hgb concentration.

## Introduction

In Ethiopia, many HIV positive patients and those co-infected with tuberculosis, sexually transmitted infections, and other opportunistic infections currently do not have reliable access to diagnostic laboratory tests. CD4 T-cell counting is the most predictable indicator of HIV disease progression, is positively linked to long-term survival rates, and is more reliable than symptomatic staging [1,2,3]. According to the Ethiopian ART national guidelines which have been revised with a supplement after the country’s adoption of WHO’s “Test and treat”, CD4 testing is required for all patients at baseline and to determine eligibility and follow up of opportunistic infection prophylaxis as well as for monitoring immunologic failure where viral load monitoring is not accessible/possible [4].

Most of CD4+ T-cell and Hgb concentration testing are primarily available at centralized laboratories in major urban centers, which are not sufficient to provide this necessary test for all patients. Patients who do not have access to reliable CD4+ T-cell testing often devote a full day of travel for each health facility visit, including sample collection and result delivery, with the loss of travel costs and man-hours. In addition, long turn-around times for tests sent away to central laboratories can delay clinical decisions and put considerable burden on patients. Additionally, blood samples generally have a short period of sample stability, thus adding further complexity to a challenged network. Finally, instrument break-downs and limited capacity at central laboratories often put a strain on the number of tests that can be run, leading to testing backlogs [5].

To overcome problems associated with centralized testing, it is possible to build new laboratories or upgrade existing laboratories. However, the cost and time required to carry this out is substantial and could significantly delay the current expansion of treatment services to those most in need. It may also be difficult to find adequately trained technical staff to operate traditional laboratory instruments in many rural areas [5, 6, 7].

There are growing numbers of high quality point of care (POC) diagnostic technologies available and there is increasing interest in using these technologies to alleviate critical testing needs without building sophisticated laboratories [8]. Pima™ POC CD4 (Alere) is the first POC technology that has been in use in Ethiopia since 2013. BD FACSPresto™ (BD Biosciences) CD4 POC technology which has prequalification by the World Health Organization (WHO) [9] might be used an alternative choice for CD4+ T-cell, CD4 percentage, and Hgb concentration testing in Ethiopia. Providing greater access to CD4 testing using POC technologies, such as the BD FACSPresto™, may be essential to maintain and increase ART coverage, early ART initiation, and reduce pre-ART patient loss to follow up [10, 11]. The aim of this study is to report on the outcomes of the performance of the BD FACSPresto™ analyser in the health facilities setting compared to the gold standard BD FACSCalibur and Sysmex XT-1800i™.

## Methods

### Study design and study population

Both venous and capillary blood samples were collected from each of 325 patients enrolled at four health centers (Burayu HC, Sendafa HC, Kirkos HC and Meshualekia HC) in Addis Ababa and Finfine zuria, between June 16 and July 10, 2015. The study participants were of both sexes and all adults (18 to 65 years of age). The CD4+ T-cell count, CD4%, and hemoglobin (Hgb) were tested at one of the four study health facilities using capillary blood and BD FACSPresto™ (Becton Dickinson, San Jose California, USA) by laboratory technicians. Venous blood samples were collected and stored at room temperature and transported to the Ethiopian Public Health Institute (EPHI), the national HIV reference laboratory, within eight hours of sample collection. According to the manufacturer, 48 hours of draw is considered as the stability time of the anti-coagulated blood stored at room temperature (20°C–25°C); while the stained sample with monoclonal antibodies should be analyzed within 24 hours [12]. At EPHI, the above tests were done using venous blood with the BD FACSPresto™, BD FACSCalibur™ (Becton Dickinson, San Jose California, USA) and for Hgb with Sysmex XT-1800i™ (Sysmex Corporation, Sysmex America, Inc USA) by laboratory technicians.

### Study procedure

All operators of the BD FACSPresto™ were trained on finger stick and venous blood collection and on operating BD FACSPresto™ for three days before the study was started.

After patient consent, testing was performed using capillary blood samples tested on the BD FACSPresto at the study site according to the manufacturer’s instructions [13]. An additional blood sample was taken by venipuncture and transported to EPHI reference laboratory for further testing using the BD FACSPresto™, the BD FACSCalibur™ and Sysmex XT-1800i™, according to manufacturers’ instructions [13, 14, 15]. The BD FACSCalibur™ and Sysmex XT-1800i™ were used as reference for CD4 T-cell counting both absolute and % values, and Hgb concentration respectively. Operators of the BD FACSCalibur™ and Sysmex XT-1800i™ were previously trained on operating FACSCalibur™ and full automated Sysmex XT-1800i™; and they had seven years of operating experience. CD4 T-cell counting was done using single platform BD Trucount™ tube with a calibrated number of fluorescent beads and monoclonal antibodies (MultiTest CD3/CD8/CD45/CD4 which contains FITC-labeled CD3, PE-labeled CD8, PerCP-labeled CD45 and APC-labeled CD4).

To prevent any potential bias, operators were blinded of the test results from the BD FACSPresto™ at the health center and tests performed at EPHI. Different study staff was responsible for the BD FACSPresto™, the BD FACSCalibur™, and Sysmex XT-1800i™ testing at EPHI and separate data entry forms were completed for the different testing platforms. Patient data and test results from the study sites were collected and transcribed in an Excel database manually once a week using double entry by two data clerks at the reference laboratory to minimize possible source of error.

The BD FACSPresto™ was also evaluated in terms of operational characteristics. Seven operators of the BD FACSPresto™ at the facilities and EPHI were selected randomly from 16 trained operators. The questionnaire included questions on the ease of use, additional supplies/equipment required, training need, time taken to perform the test compared to manufacturer’s claim and clarity of the instructions.

### Quality assurance

Prior to the start of the study, manufacturer engineers serviced and validated the BD FACSCalibur™, the BD FACSPresto™ and Sysmex XT-1800i™ devices. Performance of the BD FACSCalibur™ reference method was monitored daily using BD calibration beads (FITC, PE, PerCP and APC bead fluorescent color) to adjust instrument settings including to set instrument fluorescence and test sensitivity. Prior to sample analysis each day, single platform Trucount™ bead controls were used to monitor the number of fluorescent beads in the BD Trucount™ tube and BD Multi-check process controls were run to control the whole process starting from sample pipetting to sample acquisition. Sysmex XT-1800i™ was also calibrated and quality control samples were run prior to sample testing each day. In addition, the BD FACSCalibur™ instrument and Sysmex XT-1800i™ at the reference laboratory were enrolled in an external quality assessment (EQA) Proficiency test (PT) program, Quality Assessment Program for Standardization and immunological Measures Relevant to HIV/AIDS (QASI). The instruments passed the PT assessment during the study and during the preceding year of EQA PT panels. The BD FACSPresto™ instrument quality check was run automatically each day when the instrument was switch on. Furthermore, study site supervision was conducted weekly during the study.

### Ethical consideration

The study protocol was approved by the Scientific and Ethical Research Office (SERO) at the Ethiopian Public Health Institute, (Project number: **SERO-016-5-2015**) and all participants provided written informed consent before enrollment in the study. Only patients who provided informed consent were included in the study. For patients not willing to participate in the study, normal testing and clinical care were provided per national guidelines without negative consequences. The CD4+ test results obtained using the BD FACSCalibur™ and Hgb test results using Sysmex XT-1800i™ were sent back to the respective health center for routine care and treatment of the patient. BD FACSPresto™ test results were not given back to the patients, but remained confidential. All study materials, including hard copy results, consent and data collection forms, were kept in a locked cabinet.

### Statistical data analysis

The data were analyzed using Stata Version 11 (StataCorp LP, College Station, TX, USA), Microsoft Excel (Microsoft Corporation, Redmond, WA, USA), and GraphPad Prism (GraphPad Software Inc, La Jolla, CA, USA). The performance of the BD FACSPresto™ compared to the BD FACSCalibur™ and/or Sysmex XT-1800i™ was determined using statistical tests including median, range, standard deviation and coefficient of variation. We calculated the coefficient of variation (CV) for instrument precision, intra-assay, and inter-instrument variations for CD4+ T-cell and CD4% counting.

The regression coefficient (r^2^) between the BD FACSPresto™, the BD FACSCalibur™ and/or Sysmex XT-1800i™was analyzing using a passing and Bablok regression. The Bland-Altman (difference plot) analysis was used to determine the mean bias and 95% limit of agreement (LOA = Mean bias ±1.96 SD) [16]. For this, the difference between data pairs was graphically represented on the Y-axis against their mean on the X-axis [17]. Percentage similarity was also calculated (the average of the BD FACSCalibur™ or Sysmex XT-1800i™ and the BD FACSPresto™ divided by the BD FACSCalibur™ or Sysmex XT-1800i™ multiplied by 100) [18].

Misclassification probabilities [7] were determined at CD4+ T-cell thresholds of 100 cell/μl used for cryptococcal pneumonia reflex testing, 350 cells/μl the previously recommended ART initiation threshold [19] and 500 cells/μl, the 2013 WHO recommended ART initiation threshold [20] to determine the percentage of patients correctly classified using the BD FACSPresto™ result compared with the BD FACSCalibur™ classification. The performance of the BD FACSPresto™ was also assessed using sensitivity, specificity and predictive (positive and negative) values using the clinically relevant CD4 thresholds and 2x2 tables [17].

The venous blood samples were also used to determine the precision of the BD FACSPresto™ for CD4+ T-cell, CD4%, and Hemoglobin concentration. To measure the intra-assay variability (cartridge to cartridge variation including procedural errors) of the BD FACSPresto™, 12 samples were selected randomly and tested ten times each using ten new cartridges on the same BD FACSPresto™ device. Instrument precision (run-to-run variation) was determined using 15 venous blood samples. The run-to-run variation was determined using the same cartridge and the same device run ten times for a total of 150 tests. Finally, we assessed the inter-instrument variability (instrument-to-instrument variation) using eight venous samples run ten times on two different FACSPresto™ devices with the same cartridge.

## Results

### Demographic characteristics of study participants

A total of 325 participants (65 from Burayu HC, 76 from Sendafa HC, 96 from Kirkos HC and 88 from Meshualekia HC) were enrolled in the study. From the 325 samples collected, ten samples were excluded from accuracy and precision analysis: due to sample quality (two); due to invalid results by the BD FACSPresto™ using capillary blood (three); due to invalid results by the BD FACSPresto™ using venous blood (six). The demographic characteristic of study participants is shown in **Table 1**. The median age was 37 years old and 69% were female. Data were similar if disaggregated by gender (data not shown).

**Table 1:** Characteristics of study participants.

	Male	Female	Total
Number of subjects (%)	100 (31)	222 (69)	322 (100)
Median age (interquartile range)	42 (35.5–48)	35 (29–42)	37.0 (30–44)
Number of patients on ART (%)	87 (86)	198 (89)	285 (88)
Median (interquartile range) CD4 cell/μl (BD FACSCalibur™)	318 (168.5–469)	444 (299–598)	407.5 (258–555)
**CD4 category data by** BD FACSCalibur™**, n (%)**			
CD4 ≤100 cells/μl	9 (9)	3 (1.4)	12 (3.7)
100< CD4 ≤ 350 cells/μl	45 (45)	67 (30.2)	112 (34.8)
350< CD4 ≤ 500 cells/μl	27 (27)	66 (29.7)	93 (28.9)
CD4 >500 cells/μl	19 (19)	86 (38.7)	105 (32.6)

### Accuracy assessment

#### CD4+ T-cell comparison between the BD FACSPresto™ (Capillary) and BD FACSCalibur™

**Fig 1:**
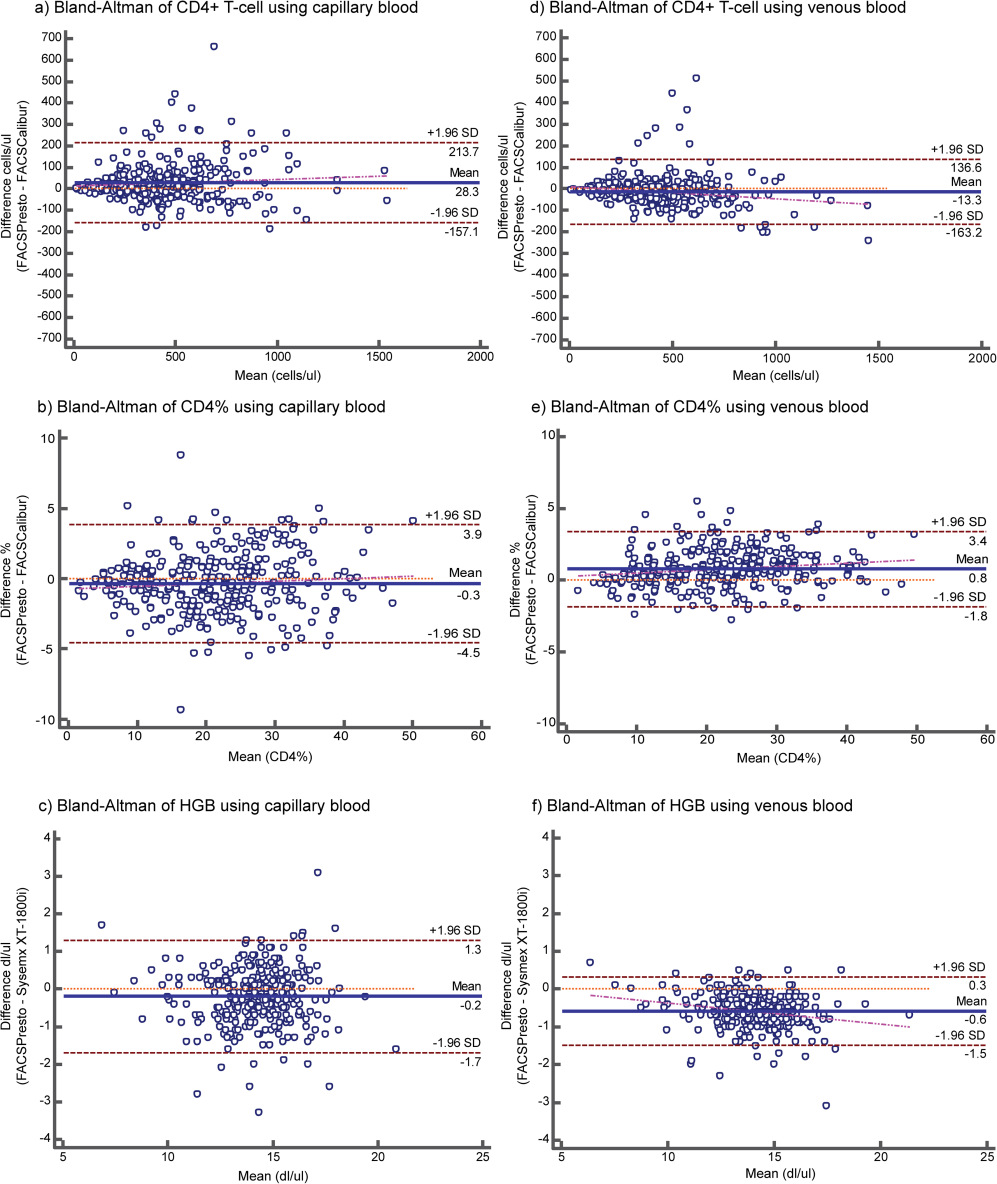
Bland-Altman comparisons between the BD FACSPresto™ with capillary blood (a-c) and venous blood (d-f) samples with the BD FACSCalibur™ reference standard. The corresponding graphs show the absolute bias between the FACSPresto™ and FACSCalibur represented in the Bland-Altman plots for CD4 T-cell testing with capillary blood (a) and venous blood (d); Bland-Altman plots for CD4% testing with capillary blood (b), venous blood (e) samples; Bland-Altman plots for Hgb testing with capillary blood (c), venous blood (f)., The solid green lines represent the mean bias and the solid deep red lines represent the upper and lower limits of agreement (LOA = mean ± 1.96SD).

**Fig 2:**
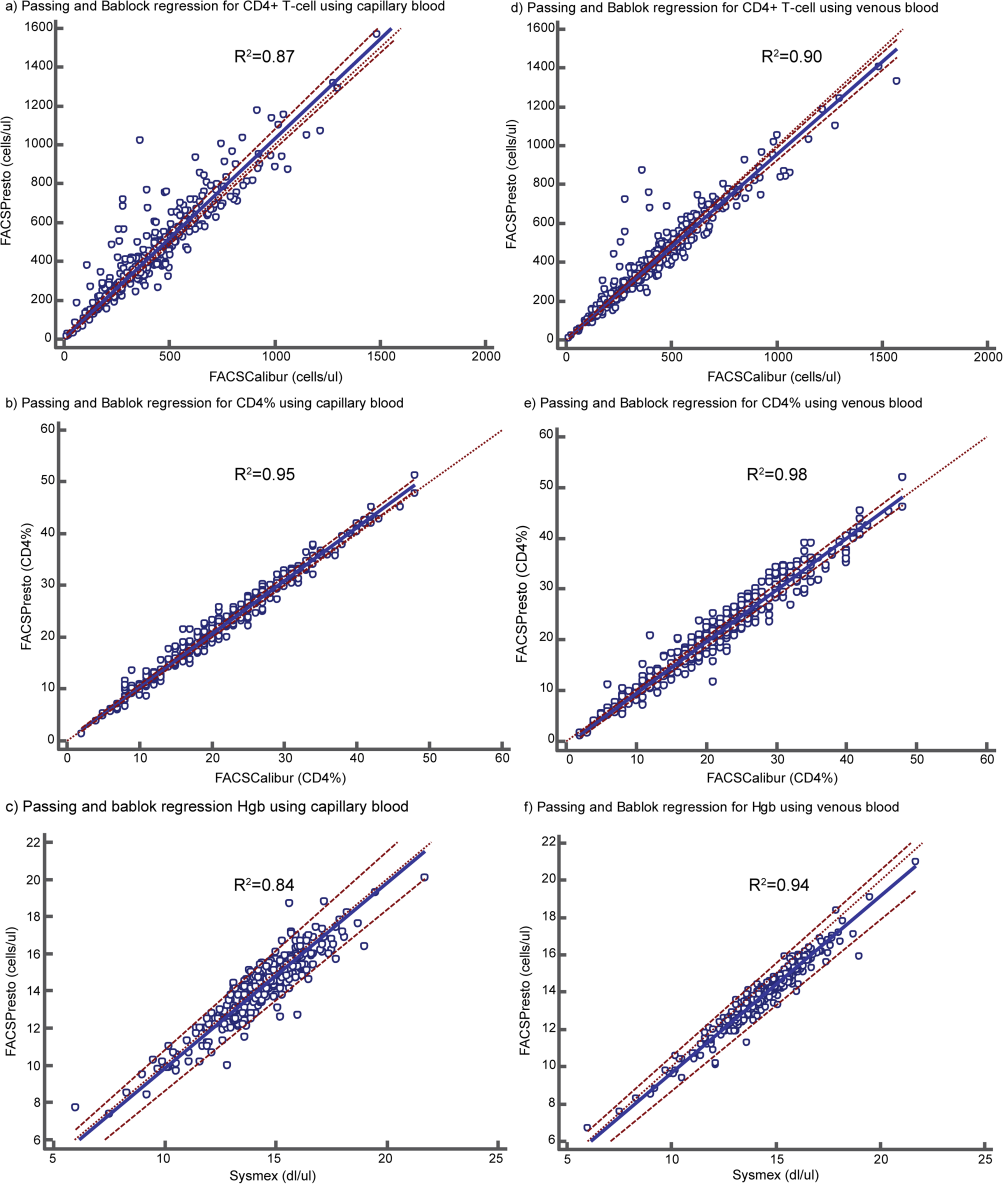
Passing and Bablok regression between the BD FACSPresto™ with capillary blood (a-c) and venous blood (d-f) compared to the BD FACSCalibur™. Comparison of the BD FACSPresto™ with BD FACSCalibur™ for CD4+ T-cell count with capillary blood (a) and with venous blood samples (d). Comparison of the BD FACSPresto™ with BD FACSCalibur™ for CD4% with capillary blood (b) and with venous blood samples (e). Comparison of the BD FACSPresto™ with Sysmex XT-1800i™with capillary blood (c) and with venous blood (f).

The median CD4+ T-cell count using the BD FACSPresto™ with capillary (finger prick) blood and the BD FACSCalibur™ using venous blood was described in **Table 2**. The BD FACSPresto™ with capillary blood had an absolute mean bias of 28.3 cells/μl (6.4%) (95% LOA: -157.1, 213.7) when compared to the BD FACSCalibur™ (**Table 2, Fig 1A)**. The BD FACSPresto™ using capillary blood and CD4 category shown in **Table 1** had an absolute mean bias of 10.3, 13.6, 3.3 and 2.4 cells/μl respectively, compared to the BD FACSCalibur™ (**S1 Table & S1A, S1B, S1C and S1D Fig**). The coefficient of determination (R^2^) between the BD FACSPresto™ with capillary blood and the BD FACSCalibur™ was 0.87 (**Fig 2A**). The mean percentage similarity and coefficient of variance (%CV) between the BD FACSPresto™ using capillary blood and the BD FACSCalibur™ was 105.2% and 15.1% respectively.

**Table 2:** FACSPresto™ comparing with BD FACSCalibur™ on the CD4+ T-cell testing.

	BD FACSPresto™ Capillary vs BD FACSCalibur™ (Venous)	BD FACSPresto™ Venous vs BD FACSCalibur™ (Venous)	BD FACSPresto™ Capillary vs BD FACSPresto Venous
N	320	317	315
CD4 cell/μl, BD FACSPresto™ (median, interquartile range)	431 (276–603.5)	407 (262–553)	
CD4 cell/μl BD FACSCalibur™ (median, interquartile range)	413.5 (260–556)	420 (263–557)	
Coefficient of determination R2	0.87	0.90	0.90
Absolute mean bias (cell/μl) (LOA)	28.3 (-157.1,213.7)	-13.3 (-163.2, 136.6)	41.7 (-94.1,177.5)
Relative bias (%) (LOA)	6.4 (-35.7, 48.6)	-2.99 (-36.7, 30.7)	9.5 (-21.9, 41.4)
Percentage of similarity, (% CV)	105.2 (15.1%)	99.3 (10.7%)	106.2 (11.3%)

Using the threshold of 100 cells/μl for cryptococcal pneumonia reflex testing, the sensitivity and specificity of the BD FACSPresto™ using capillary blood were 83.3and 99.7%, respectively, compared to the BD FACSCalibur™. The total misclassification rate of the BD FACSPresto™ with capillary blood at this threshold was 0.9% (**Table 3**).

**Table 3:** Sensitivity, specificity, upward and downward misclassification, positive and negative predictive value of the BD FACSPresto™ using capillary blood compared with the BD FACSCalibur™ reference technology across three CD4+ T cells thresholds values.

CD4+Tcell Threshold (cells/μl)	Sensitivity (95%CI)	Specificity (95%CI)	Upward misclassification n/N (%)	Downward misclassification n/N (%)	Total Misclassification n/N (%)	Positive predictive value (95%CI)	Negative predictive value (95%CI)
100	83.3% (51.6–97.9%)	99.7% (98.2–100%)	2/12 (16.7%)	1/308 (0.3%)	3/320 (0.9%)	90.9% (58.7–99.8)	99.4% (97.7–99.9)
350	82.9% (75.0–89.0)	92.9% (88.4–96.1)	21/123 (17.1%)	14/197 (7.1%)	35/320 (10.7%)	87.9% (80.6–93.2)	89.7% (84.7–93.5)
500	87.9% (82.8–91.9)	91.4% (84.5–96.0)	26/215(12.1%)	9/105 (8.6%)	35/320 (10.9%)	95.5% (91.5–97.9)	78.7% (70.4–85.6)

Using the Previous WHO 2010 ART initiation threshold of 350 cells/μl, the sensitivity and specificity of the BD FACSPresto™ with capillary blood were 82.9 and 92.9% respectively, compared to the BD FACSCalibur™. The total misclassification rate of the BD FACSPresto™ with capillary blood at this threshold was 10.7% (**Table 3**).

Using the WHO 2013 ART eligibility threshold of 500 cells/μl, the sensitivity and specificity of the BD FACSPresto™ with capillary blood were 87.9 and 92.9% respectively, compared to the BD FACSCalibur™. The total misclassification rate of the BD FACSPresto™ with capillary blood at this threshold was 10.9% (**Table 3**).

#### CD4% comparison between the BD FACSPresto™ (Capillary) and BD FACSCalibur™

The BD FACSPresto™ with capillary blood had an absolute mean bias of -0.3% (-0.1%) (95% LOA: -4.5, 3.9) compared to the BD FACSCalibur™ (**Table 4, Fig 1B**). The BD FACSPresto™ with capillary samples had an R^2^ of 0.95 compared to the BD FACSCalibur™ (**Fig 2B**).

**Table 4:** BD FACSPresto™ comparing with BD FACSCalibur™ on the CD4% testing.

	BD FACSPresto™ Capillary vs BD FACSCalibur™ (Venous)	BD FACSPresto™ Venous vs BD FACSCalibur™ (Venous)	BD FACSPresto™ Capillary vs BD FACSPresto Venous
N	320	317	315
CD4%, BD FACSPresto™ (median, interquartile range)	21.0 (14.2–28.0)	22.7 (15.7–28.7)	
CD4% BD FACSCalibur™ (median, interquartile range)	22 (15–27)	22 (15–27)	
Coefficient of determination R^2^	0.95	0.98	0.95
Absolute mean bias (LOA)	-0.3 (-4.5, 3.9)	0.8 (-1.8, 3.4)	-1.1 (-3.0,5.3)
Relative bias (%) (LOA)	-0.1 (-4.5, 3.9)	0.2 (-1.8, 3.4)	-0.3 (-5.3, 3.0)
Percentage of similarity, (% CV)	99.1 (6.4%)	101 (4.2%)	97.4 (5.5%)

#### Hemoglobin (Hgb) comparison between the BD FACSPresto™ (Capillary) and Sysmex XT-1800i™

The absolute mean bias of Hgb concentration using the BD FACSPresto™ with capillary blood was -0.2 dl/μl (95% LOA: -1.7, 1.3) compared to the Sysmex XT-1800i™ (**Table 5 & Fig 1C**). Hgb concentration testing using the BD FACSPresto™ with capillary samples had an R^2^ of 0.84 compared to the Sysmex XT-1800i™ (**Fig 2C**). The mean percentage similarity and %CV between the BD FACSPresto™ with capillary blood and the Sysmex XT-1800i™ was 94.4% and 2.7% respectively (**Table 5**).

**Table 5:** FACSPresto comparing with Sysmex XT-1800i™on the Hemoglobin concentration testing.

	BD FACSPresto™ Capillary vs Sysmex XT-1800i™ (Venous)	BD FACSPresto™ Venous vs Sysmex XT-1800i™ (Venous)	BD FACSPresto™ Capillary vs BD FACSPresto Venous
N	321	319	318
Hgb dl/μl, BD FACSPresto™ (median, interquartile range)	14.3 (13–15.3)	13.9 (12.8–14.8)	
Hgb dl/μl Sysmex XT-1800i™ (median, interquartile range)	14.4 (13.4–15.5)	14.4 (13.4–15.5)	
Coefficient of determination R2	0.84	0.94	0.84
Absolute mean bias (dl/μl) (LOA)	-0.2 (-1.7, 1.3)	-0.59 (-1.49, 0.31)	-0.4 (-1.8,1.0)
Relative bias (%) (LOA)	0.0 (-1.7, 1.3)	-0.1 (-1.5, 0.3)	0.1 (-1.0, 1.8)
Percentage of similarity, (% CV)	99.4 (2.7%)	98 (1.6%)	101.5 (2.7%)

#### CD4+ T-cell comparison between the BD FACSPresto™ (venous blood) and BD FACSCalibur™

The BD FACSPresto™ with venous blood had an absolute mean bias of -13.3 cells/ul (-2.99%) (95% LOA: -163.2, 136.6) compared to the BD FACSCalibur™ (**Table 2 & Fig 1D**). The BD FACSPresto™ using capillary blood at CD4 category (**Table 1**) had an absolute mean bias of -17.8, 0.3, 3.6 and -5.7 cells/μl respectively, compared to the BD FACSCalibur™ (**S1 Table & S1F, S1G and S1H Fig**). The BD FACSPresto™ with venous blood had an R^2^ of 0.90 compared to the BD FACSCalibur™ (**Fig 2D**). The mean percentage similarity and %CV between the BD FACSPresto™ with venous blood and the BD FACSCalibur™ were 99.3% and 10.7%, respectively.

The sensitivity and specificity at the 100 cells/μl threshold of the BD FACSPresto™ with venous blood were 100 and 99.4% respectively, compared to the BD FACSCalibur™. The total misclassification rate of the BD FACSPresto™ with venous blood at this threshold was 0.63% (**Table 6**).

**Table 6:** Sensitivity, specificity, upward and downward misclassification, positive and negative predictive value of the BD FACSPresto™ using venous blood compared with the BD FACSCalibur™ reference technology across three CD4+ T cells thresholds values.

CD4+Tcell Threshold cells/μl	Sensitivity (95%CI)	Specificity (95%CI)	Upward misclassification n/N (%)	Downward misclassification n/N (%)	Total Misclassification n/N (%)	Positive predictive value (95%CI)	Negative predictive value (95%CI)
100	100% (59.0–100%)	99.4% (97.7–99.9)	0/7 (0.0%)	2/310 (0.65%)	2/317 (0.63%)	77.8% (40–97.2)	100% (98.8–100.0)
350	91.6% (85.0–95.9)	91.9% (87.2–95.3)	10/119 (8.4%)	16/198 (8.1%)	26/317 (8.2%)	87.2% (80.0–92.5)	94.8% (90.6–97.5)
500	94.3% (90.3–97.0)	83.8% (75.3–90.3)	12/212 (5.7%)	17/105 (16.2%)	29/317 (9.2%)	92.2% (87.8–95.4)	88.0% (80.0–93.6)

Using the previous WHO ART eligibility threshold of 350 cells/μl, the sensitivity and specificity of the BD FACSPresto™ with venous blood were 91.6 and 91.9% respectively, compared to the BD FACSCalibur™. The total, upward and downward misclassification rates of the BD FACSPresto™ with venous blood at this threshold were 8.2, 8.4 and 8.1% respectively (**Table 6**).

Using the 2013 WHO ART eligibility threshold of 500 cells/μl, the sensitivity and specificity of the BD FACSPresto™ with venous blood were 94.3 and 83.8% respectively, compared to the BD FACSCalibur™. The total, upward and downward misclassification rates of the BD FACSPresto™ with venous blood at this threshold were 9.2, 5.7% and 16.2%, respectively (**Table 6**).

#### CD4% comparison between the BD FACSPresto™ (venous blood) and BD FACSCalibur™

The BD FACSPresto™ with venous blood had an absolute mean bias of 0.8 (0.2%) (95% LOA: -1.8, 3.4) (**Fig 1E**) compared to the BD FACSCalibur™. The BD FACSPresto™ with venous blood had an R^2^ of 0.98 compared to the BD FACSCalibur™ (**Fig 2E**). The mean percentage similarity and %CV between the BD FACSPresto™ with venous blood and the BD FACSCalibur™ were 98% and 1.6%, respectively (**Table 4**).

#### Hemoglobin (Hgb) comparison between the BD FACSPresto™ (venous blood) and Sysmex XT-1800i™

For Hgb concentrations testing, the BD FACSPresto™ with venous blood had an absolute mean bias of -0.59 dl/μl (-0.1%) (95% LOA: -1.49, 0.31) (**Fig 1F**) compared to the Sysmex XT-1800i™. The BD FACSPresto™ with venous blood had an R^2^ of 0.94 compared to the Sysmex XT-1800i™ (**Fig 2F**). The mean percentage similarity and the %CV between the BD FACSPresto™ with venous blood and Sysmex XT-1800i™were 98% and 1.6%, respectively (**Table 5**).

#### CD4+ T-cell comparison between the BD FACSPresto™ (capillary) and BD FACSPresto™ (venous)

The CD4+ T-cell count of BD FACSPresto with capillary blood had an absolute mean bias of 41.7 cells/μl (9.5%) (95% LOA: -94.1, 177.5) compared to BD FACSPresto™ with venous blood (**Table 2**). The BD FACSPresto™ had an R^2^ of 0.90 with capillary blood compared to the BD FACSPresto™ with venous blood. The mean percentage similarity and %CV between the BD FACSPresto™ using capillary and venous blood were 106.2% and 11.3%, respectively (**Table 2**).

#### CD4% count comparison between the BD FACSPresto™, capillary and BD FACSPresto™, venous

The absolute mean bias of the CD4% using the BD FACSPresto™ comparing capillary and venous blood was -1.1(0.3%) (95% LOA: -3.0, 5.3). The BD FACSPresto™ with capillary blood had an R^2^ of 0.95 compared to the BD FACSPresto™ with venous blood (**Table 4**).

#### Hgb concentration comparison between the BD FACSPresto™ (capillary) and BD FACSPresto™ (venous)

The mean bias of the Hgb concentration using the BD FACSPresto™ with capillary blood was -0.1dl/μl (0.1%) (95% LOA: -1.8, 1.0) compared to the BD FACSPresto™ with venous blood. The BD FACSPresto™ with capillary blood had an R^2^ of 0.84 compared to the BD FACSPresto™ (**Table 5**).

#### Precision assessment of the BD FACSPresto™

Intra-assay variability was determined by repeating 12 different samples with CD4+ T-cell counts between 100–500 cells/μl (**Table 7**). The mean %CV of intra-assay of absolute CD4+ T-cell, CD4% and Hgb concentration was 6.2, 5.2, and 2.2% respectively. We also measured the precision of CD4+ T-cell, CD4% and Hgb concentration results using the same sample run on two different BD FACSPresto™ devices to evaluate the inter-instrument variation. The mean %CV of the CD4+ T-cell count, CD4% and Hgb concentration was 6.5, 5.7 and 1.7% respectively.

**Table 7:** Precision of the BD FACSPresto™ technology.

Precision (Variability)	# of samples	Repeating test for each sample	Average %CV (range) for CD4+ T-cell count	Average %CV (range) for CD4% count	Average %CV (range) for Hgb concentration
Intra-Assay	12	10 replicate	6.2% (3.5–12.3)	5.2% (2.6–11.2)	2.2% (1.1–3.3)
Inter-Instrument	8	10 replicate	6.5% (3.7–13.2)	5.7% (2.8–11.4)	2.4% (1.3–3.9)
Instrument precision	15	10 runs	4.2% (1.2–10.7)	3.5% (1.1–9.8)	1.7% (0.9–4.9)

The run-to-run instrument precision using the BD FACSPresto™ for CD4+ T-cell count, CD4% count and Hgb concentration had a mean %CV of 4.2, 3.5 and 1.7% respectively.

#### Error rates of FACSPresto™

A total of three (0.9%) samples out of the 325 samples produced an error when tested on the BD FACSPresto™ using capillary blood in the health facility. One of the three samples produced a valid result after repeat testing with the same cartridge. Six samples out of the total 323 (1.9%) tested on the BD FACSPresto™ using venous blood at the reference laboratory produced an error. Three of the six samples produced a valid result after repeat testing with a new cartridge using the same venous sample (**Table 8**).

**Table 8:** Error rates of the BD FACSPresto™ technology.

Characteristics	BD FACSPresto™ Capillary	BD FACSPresto™ Venous
Total tested (N)	325	323
No of valid Result obtained	322	317
No of invalid result obtained (n)	3	6
Failure rate %, (n/N)	0.9 (3/325)	1.9 (6/323)

#### Operational characteristics assessment

The operational characteristics of the BD FACSPresto™ were assessed by interviewing seven laboratory technicians who operated the BD FACSPresto™ both at the health centers and the reference laboratory using a standard questionnaire. All of the operators responded that the BD FACSPresto™ was very simple to use and instrument instructions were concise and clear. Six of seven (86%) laboratory technicians responded that one day was the minimum number of days required for training to operate the BD FACSPresto™, where as one operator responded that three days of training was preferred.

## Discussion

This independent technical accuracy study was conducted to assess the performance of the BD FACSPresto™ technology to accurately quantify CD4+ T cells, CD4% count, and Hgb concentration using capillary and venous blood from adult HIV patients. The performance of the BD FACSPresto™ to enumerate CD4+ T cells and CD4% was comparable to the laboratory-based BD FACSCalibur™, while the performance of the BD FACSPresto™ to quantify Hgb concentration was comparable to the laboratory-based Sysmex XT-1800i™. The BD FACSPresto™ had a sensitivity of over 90% and ≤90% to detect patients below the three thresholds measured using venous and capillary blood respectively: 100 cells/μl used for reflex Cryptococcal testing, 350 cells/μl previous ART initiation and 500 cells/μl ART failure thresholds. The precision of the BD FACSPresto™ was strong with all precision measurements having a coefficient of variation of less than 7%. Finally, the test error rates were very low, both in the health centers and in the reference laboratory. It is also easy to operate FACSPresto™ for testing.

Though the performance of the BD FACSPresto™ was comparable to the laboratory assay using venous samples, some over-quantification by the BD FACSPresto™ was observed when capillary blood samples were tested. Over 10% of patients were incorrectly classified as above each of the included CD4+ T cell thresholds, suggesting that they would not have received the required reflex cryptococcal testing. These results are consistent with those previously observed [21]. Upward misclassifying a proportion of patients would result in lower costs of testing and treatment; however, costs may not be lower in a global perspective (due to complications, not only a lack of well-being), so "direct costs" should rather be used. National programs, however, may be more willing to perform additional cryptococcal tests and initiate patients just above the ART eligibility thresholds for improved patient care and outcomes. In that setting, a technology that is more comparable to the laboratory assay or tends towards under-quantification and downward misclassification may be more appropriate for reducing patient morbidity and mortality.

While the BD FACSPresto™ performed more comparably using venous samples with reduced upward misclassification rates, some point-of-care settings require capillary blood samples when phlebotomist and pipetting skills are lacking. Being able to utilize both sample types will provide significantly more flexibility in product placement considerations and allow for further decentralization where necessary. Countries may want to consider these results as they plan strategic deployment.

Ethiopia has an extensive laboratory network, including conventional laboratory-based CD4+ T cell testing. Unfortunately, this laboratory network does not reach all patients in need of CD4+ T cell testing with many lacking on-site testing. Like Pima^TM^, Point-of-care technologies, such as the BD FACSPresto™, could support increased access to on-site CD4+ T cell testing, particularly in rural and hard to reach settings. Thus, alternative deployment strategies are being considered in Ethiopia and other countries. In Ethiopia, BD FACSCount technologies have been in use for many years; however, device breakdowns have rendered several inoperable. Given its high throughput, rather than procuring new BD FACSCount technologies, replacing those not functioning with BD FACSPresto™ technologies is being considered to be more beneficial. Compared to the BD FACSCount, the BD FACSPresto™ does not require laboratory or pipetting skills, refrigeration of reagents and controls, consistent electricity, or additional procurement of controls and yet has a similar test turnaround time.

The 2016 WHO ART Consolidated Guidelines recommend treating all patients with ART regardless of the CD4+ T cell count [22]. Implementing this recommendation could be impactful for countries in improving patient lives, reducing transmission, and achieving the UNAIDS’ 90-90-90 targets [23, 24]. High HIV burden countries in sub-Saharan Africa are left with difficult decisions of when and how to implement the many new recommendations in resource-limited settings. In Ethiopia, although, the guideline has been adopted, financial constraints persist that will not allow immediate uptake of treat all. In the interim, CD4+ T cell counting will remain a critical test to prioritize patients in most need of ART. Furthermore, CD4+ T cell testing is an important diagnostic in supporting clinical opportunistic infection monitoring of all HIV-positive patients.

In accordance with the 2013 WHO ART Consolidated Guidelines, Ethiopia initiates infants under five years of age on ART regardless of CD4%. While this test is no longer a barrier to ART, clinicians still find it useful in understanding the immunological status of infants prior to initiation and monitoring. Furthermore, Hgb testing remains critical for patients initiating ART using zidovudine (AZT) as the drug can cause hematological toxicity, particularly in patients with low body weight, low CD4+ T cell count, and/or advanced disease. Unfortunately, access to Hgb testing in Ethiopia is minimal; therefore, this multiplex BD FACSPresto™ technology would allow for increased access to CD4+ T cell and Hgb testing for adults as well as CD4% testing for infants.

Several limitations exist in this study. First, diagnostic testing is primarily performed by trained laboratory staff in Ethiopia. While this is consistent across the country, point-of-care technologies are often operated by non-laboratory staff in other countries. Understanding the performance of these technologies when used by non-laboratory staff would be very helpful in countries considering task-shifting. Additionally, the confidence intervals for some of the clinical comparisons (sensitivity, specificity, and misclassification) were quite wide. This was primarily due to the lower performance of the BD FACSPresto™ to correctly classify patients above or below the thresholds analyzed. Patient sample sizes were sufficiently calculated based on higher performance expectations. Though this technical evaluation took place in Addis Ababa and the surrounding area, it is not expected that the performance of the BD FACSPresto™ technology would vary if placed in a rural setting and if used for measuring CD4% in infants. Although Pima™ has been deployed for about three years in Ethiopia; this study did not compare BD FACSPresto™’s performance with this POC CD4. However, the study by Bwana et al has demonstrated their similarity [21].

In conclusion, this study highlighted comparable performance of the BD FACSPresto™ to the BD FACSCalibur™ for CD4+ T cell and CD4% enumeration and the Sysmex XT-1800i™ for Hgb counting. The BD FACSPresto™, in combination with rapid HIV diagnostic tests, provides an opportunity to rapidly diagnose and assess ART eligibility in a single visit. Furthermore, patients with more advanced disease can be prioritized for immediate follow-up and rapid ART initiation. Thus, the BD FACSPresto™ is a potential addition to the CD4 testing network to both assess ART eligibility and support opportunistic infection monitoring.

## Supporting information

S1 Fig Bland-Altman comparisons between the BD FACSPresto™ with capillary blood (a-d) and venous blood (e-h) samples with the BD FACSCalibur™ reference standard. The corresponding graphs show the absolute bias between the FACSPresto™ and FACSCalibur at CD4≤100 cells/μl represented in the Bland-Altman plots for CD4 T-cell testing with capillary blood (a) and venous blood (e); Bland-Altman plots for CD4 absolute between 100 and 350 cells/μl category with capillary blood (b), venous blood (f) samples; Bland-Altman plots for CD4 absolute between 350 and 500 cells/μl category testing with capillary blood (c), venous blood (g); Bland-Altman plots for CD4>500 cells/μl category testing with capillary blood (d), venous blood (h). The solid green lines represent the mean bias and the solid deep red lines represent the upper and lower limits of agreement (LOA = mean ± 1.96SD).
(TIF)

S1 Table BD FACSPresto™ comparing with BD FACSCalibur™ based on the CD4+ T cell category. Absolute mean bias was compared based on at CD4≤100 cells/μl, between 100 and 350 cells/μl, between 350 and 500 cells/μl and CD4>500 cells/μl category testing.
(DOC)

S1 Data The demographic characteristic, CD4 T-cell, CD4% and Hgb values using BD FACSPresto™ and BD FACSCalibur™ done at national laboratory and site for each study participants which coded by patient study ID. Error result given by FACSPresto at site and national laboratory also included.
(XLS)
