# Supplementary figures and images for: The performance of BD FACSPresto™ for CD4 T-cell count, CD4% and hemoglobin concentration test in Ethiopia
Source: PLoS One. 2017 Apr 27;12(4):e0176323. doi: 10.1371/journal.pone.0176323 (PMC5407647; doi:10.1371/journal.pone.0176323)

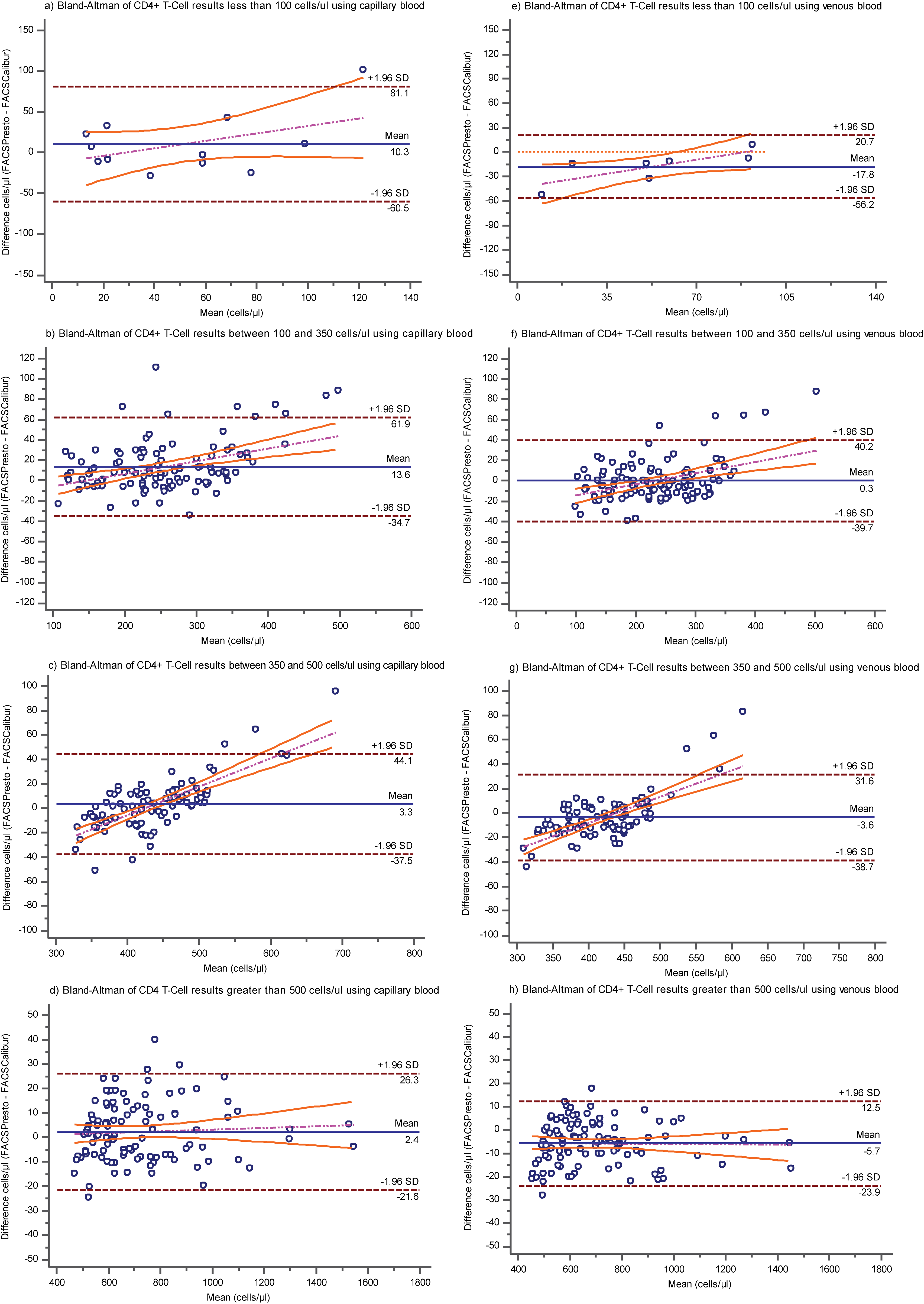

Supplement: S1 Fig — The corresponding graphs show the absolute bias between the FACSPresto™ and FACSCalibur at CD4≤100 cells/μl represented in the Bland-Altman plots for CD4 T-cell testing with capillary blood (a) and venous blood (e); Bland-Altman plots for CD4 absolute between 100 and 350 cells/μl category with capillary blood (b), venous blood (f) samples; Bland-Altman plots for CD4 absolute between 350 and 500 cells/μl category testing with capillary blood (c), venous blood (g); Bland-Altman plots for CD4>500 cells/μl category testing with capillary blood (d), venous blood (h). The solid green lines represent the mean bias and the solid deep red lines represent the upper and lower limits of agreement (LOA = mean ± 1.96SD). (TIF) [file pone.0176323.s001.tif]
