# Supplementary material for: The performance of BD FACSPresto™ for CD4 T-cell count, CD4% and hemoglobin concentration test in Ethiopia
Source: PLoS One. 2017 Apr 27;12(4):e0176323. doi: 10.1371/journal.pone.0176323 (PMC5407647; doi:10.1371/journal.pone.0176323)
Supplement: S1 Table — Absolute mean bias was compared based on at CD4≤100 cells/μl, between 100 and 350 cells/μl, between 350 and 500 cells/μl and CD4>500 cells/μl category testing. (DOC) [file pone.0176323.s002.doc]

**S1 Table**: BD FACSPresto™ comparing with BD FACSCalibur™ based on the CD4+ T cell category

|  | | |
| --- | --- | --- |
| CD4+ T-cell category | BD FACSPresto™ Capillary vs BD FACSCalibur™ (Venous) | BD FACSPresto™ Venous vs BD FACSCalibur™ (Venous) |
|  | absolute mean bias (cells/µl) [95% limit of agreement (LOA)] | absolute mean bias (cells/µl) [95% limit of agreement (LOA)] |
| CD4 ≤100 cells/μl | 10.3 (-60.5, 81.1) | -17.8 (-56.2, 20.7) |
| 100< CD4 ≤ 350 cells/μl | 13.6 (-34.7, 61.9) | 0.3 (-39.7, 40.2) |
| 350< CD4 ≤ 500 cells/μl | 3.3 (-37.5, 44.1) | 3.6 (-38.7, 31.6) |
| CD4 >500 cells/μl | 2.4 (-21.6, 26.3) | -5.7 (-23.9, 12.5) |
